# Sensitive *GATA1* mutation screening reliably identifies neonates with Down syndrome at risk for myeloid leukemia

**DOI:** 10.1038/s41375-021-01128-1

**Published:** 2021-01-22

**Authors:** Bianca F. Goemans, Sanne Noort, Marjolein Blink, Yong-Dong Wang, Susan T. C. J. Peters, Jacobus P. van Wouwe, Gertjan Kaspers, Valerie de Haas, Wouter J. Kollen, Vincent H. J. van der Velden, Tanja A. Gruber, Christian M. Zwaan

**Affiliations:** 1 Princess Máxima Center for Pediatric Oncology, Utrecht, the Netherlands; 2 Department of Pediatric oncology, Sophia Children’s hospital, Erasmus medical center, Rotterdam, the Netherlands; 3 Department of Pediatrics, Amsterdam UMC, location VUmc, Amsterdam, the Netherlands; 4 Department of Cell and Molecular Biology, St. Jude Children’s Research Hospital, Memphis, TN USA; 5 Department of Child Health, Netherlands Organization of Applied Scientific Research TNO, Leiden, the Netherlands; 6 Pediatric Oncology, Emma Children’s Hospital, Amsterdam UMC, Vrije Universiteit, Amsterdam, the Netherlands; 7 Dutch Childhood Oncology Group (DCOG), Utrecht, the Netherlands; 8 Department of Immunology, Erasmus MC, University Medical Center Rotterdam, Rotterdam, the Netherlands; 9 Department of Pediatrics, Stanford School of Medicine, Stanford, CA USA

**Keywords:** Acute myeloid leukaemia, Oncogenes, Cancer genetics

## To the Editor:

Children with Down syndrome (DS) have a higher chance of developing myeloid neoplasms as compared to healthy children [1]. Therefore, the World Health Organization (WHO) classifies myeloid neoplasms associated with DS as separate from other myeloid neoplasms. The most recent WHO classification defines transient abnormal myelopoiesis (TAM) in children with DS as “increased peripheral blood (PB) blast cells in a neonate with DS”. No minimal percentage of blasts is defined to specify “increased” [2]. Using this definition, about 5–10% of neonates with DS develop TAM [3]. Approximately 1% of patients with DS develop myeloid leukemia of DS (ML DS) between 1 and 4 years of age. Approximately 20% of children diagnosed with TAM will develop ML DS [4].

Both TAM and ML DS are associated with mutations in the *GATA1* gene [5]. Mutations in *GATA1* result in a truncated protein (GATA1s). The majority of mutations are localized in exon 2 or 3. *GATA1* mutations are patient-specific, and identical in patients diagnosed with both TAM and ML DS. TAM is usually diagnosed when the patient is ~1 week old and blasts disappear spontaneously after 4 weeks in around 80% of children [3]. Approximately 20% of children develop serious symptoms and around 10% of children with TAM die in the first months of life as a result of TAM [3]. Therefore, in some cases, treatment is indicated using low dose cytarabine. This treatment has improved the outcome of children with severe TAM [3].

The aim of this study was to define a population-based frequency of TAM in DS newborns. The Dutch Pediatric Surveillance Unit (DPSU) registers newborns with DS and TAM screening was added to this registration from Jan 1, 2008, until Jan 1, 2013 (Erasmus MC MEC-2007-168, Netherlands Trial Register NL1587). Based on the live birth prevalence of DS in the Netherlands, an estimated 1211 DS children were born in the Netherlands during the screening period. Between 2008 and 2013, 803 neonates with DS were included in the DPSU registry. Therefore ~66% of the neonates with DS were registered. Of the 803 registered neonates, TAM screening blood samples were received for 368 neonates (46% of the registered cases). The low inclusion rate could give rise to a selection bias, as pediatricians are probably more likely to include neonates with symptoms or abnormal PB values compared to healthy DS neonates. Therefore, the incidence of TAM could be overestimated in this study.

**Fig. 1:**
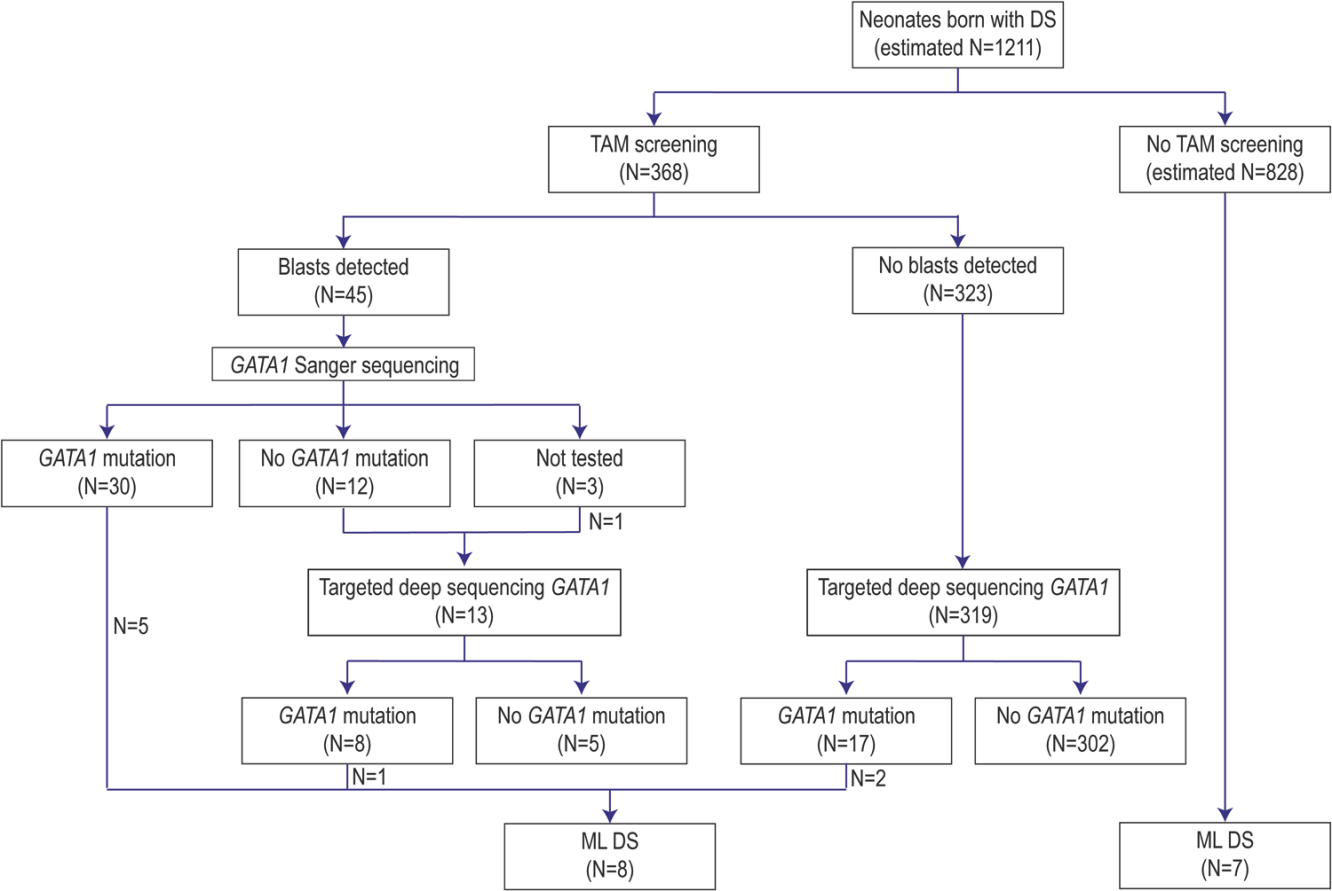
This flow chart describes the results of TAM screening in 368 Dutch newborns with Down syndrome (DS). An estimated 1158 neonates with DS were born during the study period in the Netherlands. 368 neonates were screened using peripheral blood morphology and immunophenotyping. If TAM was diagnosed, *GATA1* mutation screening was performed using Sanger sequencing (Ss). In addition, using targeted deep sequencing (TDS) as a more sensitive screening method, we retrospectively screened all TAM negative cases and all TAM cases without a *GATA1* mutation for *GATA1* mutations. All children were followed until the age of 4 years to see if they developed ML DS. TAM transient myeloproliferative disorder, ML DS myeloid leukemia of DS.

We screened 368 PB samples from DS neonates for TAM within the first 4 weeks of life using morphology and flow cytometry (technical details in Supplemental methods). The median postnatal age at screening was 5.0 days (p25–p75 0–11 days) and their clinical characteristics are detailed in Table 1. In 45 of the 368 cases screened (12%), blasts were detected (Fig. 1). The median blast percentage using morphology was 10% (range 1–91%). Of these neonates, 34 had at least 5% blasts (9.1%) and 31 had at least 10% blasts (8.4%). Six children died in the neonatal period, four due to TAM related complications, one of asphyxia, and one as a result of multiple congenital abnormalities. All children who had peripheral blasts were offered inclusion in the Flasinski study [6].

**Table 1:** The clinical characteristics of Down syndrome (DS) neonates in this study. In the “no blasts detected” column are the patients who were screened and in whom no blasts were detected using immunophenotyping and morphology, in the “blasts detected” column only those in which blasts were detected and who were classified as TAM.

	No blasts detected	Blasts detected
Total number of patients	323	45
Sex (male)	177/323 (55%)	19/36 (53%)
Median pregnancy duration (weeks, range)	38 (31–42)	37.5 (33–40)
Died in the neonatal period	3/108 (2.8%)	6/21 (29%)
First postnatal complete blood count		
Hemoglobin (mmol/L, median, p25–p75)	12.7 (10.7–13.9)	11.0 (9.4–12.4)
White blood cell count (median, *10 9/L, p25–p75)	15.1 (10.7–21)	20.1 (12.4–37.3)
Thrombocyte count (median, *10 9/L, p25–p75)	137 (85–201)	121 (40–201)
Blasts by morphology (%, median, p25–75)	0	10 (4.5–28%)
Hydrops fetalis	0	3/14 (21%)
Pleural effusion	2/170 (1.2%)	1/14 (7%)
Pericardial effusion	2/168 (1.2%)	2/14(14%)
Ascites	0	3/14 (21%)
ML DS (during follow-up)	2/323 (0.6%)	6/45 (13%)
DS ALL (during follow-up)	3/323 (0.9%)	1/45 (2.2%)

Nine children with TAM received at least one course of cytarabine (range 1–3 courses). Treatment was initiated because of clinical symptoms. Three of these nine treated children died during or shortly after treatment, all related to the TAM (two patients as a result of sepsis, one of liver failure).

In 30 of 42 neonates (71%) with detectable blasts and available DNA, a *GATA1* exon 2 mutation was detected by Sanger sequencing (Ss). As Ss can be false negative in cases with blast percentages below ~20%, targeted deep sequencing (TDS) was performed on the 12 cases with detectable blasts but without a *GATA1* mutation by Ss. Furthermore, in 1 of the 3 cases without available DNA initially, the material was found and analyzed with TDS. In 8 out of these 13 patients, a *GATA1* exon 2 mutation was detected using TDS (median mutational frequency 6.6%). No exon 3 mutations were detected. Overall, 38 of 43 neonates with blasts tested using Ss and/or TDS were positive for a *GATA1* mutation (88%).

In addition, 319 of the 323 PB samples of DS neonates without blasts, were screened for *GATA1* mutations using TDS. In four cases no material for analysis was available. In 17 cases an exon 2 mutation was detected (5.3%). Therefore, the total number of cases with a *GATA1* mutation was 55 (15%). No exon 3 mutations were detected. There was one case with 9% blasts where no *GATA1* exon 2 or 3 mutation was detected.

As the WHO does not specify the percentage of blasts to define TAM, different groups use different blast percentages ranging from ≥1% to ≥10% to define TAM in neonates with DS [6–9]. Therefore, we defined TAM as either >5% blasts (as determined by immunophenotyping or morphology), and/or detection of a *GATA1* mutation. Using this definition resulted in 34 neonates with TAM as defined by immunophenotyping and morphology (9.1%), and 55 neonates with a mutation in *GATA1* (15.2%), for a total of 56 patients with TAM. This resulted in a sensitivity of 58% and a specificity of 100% for immunophenotyping and a sensitivity of 95% and a specificity of 100% for *GATA1* screening either through Ss or TDS. One patient had 9% blasts, liver fibrosis, and severe cholestasis but we could not detect a *GATA1* mutation. No other cause of liver fibrosis was identified. The patient was treated with cytarabine but despite this treatment ultimately died. We cannot exclude that a rare *GATA1* mutation occurred outside the screened region in this patient, although this has not been previously described [10].

The recent study by Roberts et al. described a cohort of 200 hospitalized DS neonates screened for TAM and reported 17/200 TAM cases by conventional screening and an additional 18/88 “silent” TAM cases identified using NGS, leading to an overall frequency *GATA1* mutations 29% [11]. In our study, the overall frequency of *GATA1* mutations was lower, in total 15% (55/368).

Fifteen children with Down syndrome who were born in the Netherlands during the screening period were diagnosed with ML DS between 2008 and February 2019. As the DCOG registers all cases of pediatric leukemia in the Netherlands, this is a population-based number. Therefore, 1.2% (15/1211) of the neonates with DS developed ML DS. Of these 15 children, 8 had been screened for TAM after birth. Therefore, the frequency of ML DS in the screened cohort was 2.1%, this was not significantly different from the unscreened cohort (*p* = 0.07). In six cases blasts were detected after birth, and two out of these six children had symptomatic TAM treated with two courses of cytarabine. Two DS neonates later diagnosed with ML DS, had been screened but no blasts were detected. However, in both of these cases, the neonatal PB samples harbored *GATA1* exon 2 mutations detected through TDS. In the ML DS cells of these patients, exon 2 mutations identical to the mutations at birth were detected.

TAM is an important predictor of ML DS as most patients who developed ML DS in this study had peripheral blasts and all had a mutation in *GATA1* in the neonatal period. Although no preventive therapy of ML DS has been discovered until now, early detection of ML DS can help improve outcome as patients may not have developed symptoms as yet at the time of detection, and hence may be in a better general condition when they start chemotherapy. Furthermore, being able to define which neonates with DS are at risk of developing ML DS is relevant information for parents, as parents of children not fulfilling TAM criteria can be informed that the risk for ML DS is extremely low. However, the increased risk for acute lymphoblastic leukemia (ALL) in DS children still applies. Therefore, we advocate screening all DS neonates for peripheral blood blasts and *GATA1* mutations, using sensitive PCR techniques.

In light of the current study and the study by Roberts et al., we recommend updating the TAM definition in the next revision of the WHO classification. First of all, as there is no minimum number of blasts defined, this can lead to overdiagnosis, as neonates may often have benign immature cells in their PB. Furthermore, all patients who developed ML DS and were included in this study had a *GATA1* mutation in the neonatal period. Thus, we recommend to re-define TAM as “the presence of at least 5% blasts defined by immunophenotyping or morphology and/or the presence of a *GATA1* mutation in a neonate with DS.” This would lead to the identification of a group of neonates at risk for the development of ML DS who can receive close follow-up for early detection of ML DS.

## Supplementary information

Supplemental methods
