# Supplementary material for: Sensitive GATA1 mutation screening reliably identifies neonates with Down syndrome at risk for myeloid leukemia
Source: Leukemia. 2021 Jan 22;35(8):2403–6. doi: 10.1038/s41375-021-01128-1 (PMC8324475; doi:10.1038/s41375-021-01128-1)
Supplement: Supplementary file 1 — Supplemental methods [file 41375_2021_1128_MOESM1_ESM.docx]

**Supplemental Files**

**Supplemental file 1**

**Neonatal TAM screening**

Neonatal TAM screening was performed at the Dutch Childhood Oncology Group (DCOG) central lab. The samples were evaluated for the presence of TAM using standard morphology by two independent experienced morphologists from the DCOG lab and by flow cytometry using the megakaryoblastic markers CD41, CD36, CD117, CD9 and CD32, and in addition aberrant markers such as CD56 and CD7. TAM was defined as detection of blasts by morphology or flow cytometry. Left over viable cells were stored. No minimal percentage of blasts was defined to diagnose TAM. When TAM was detected, DNA was isolated using the QIAmp DNA blood mini kit (Qiagen). *GATA1* exon 2 mutations were detected using PCR-hetero-duplex analysis followed by Sanger sequencing (Ss) (supplemental file 2).

**Supplemental file 2**

***GATA1* Sanger sequencing**

*GATA1* exon 2 mutations were detected using PCR-hetero-duplex analysis followed by Sanger sequencing. Briefly, 50 pg DNA was amplified using a GATA1-specific forward primer located in intron1 (5’ AAAGGAGGGAAGAGGAGCAG 3’) and a GATA1-specific reverse primer located in intron2 (5’ GACCTAGCCAAGGATCTCCA 3’). After PCR (40 cycles of 10”94°C, 10”60°C, 60”68°C) 10 ul PCR product was used for heteroduplex analysis. Samples were denatured for 5 minutes at 94°C and subsequently cooled to 4°C for 60 minutes to induce duplex formation, which were separated in a 5% precast polyacrylamide gel. If multiple heteroduplex bands were observed, the bands were excised from the gel and sequencing was performed using the BigDye Terminator v3.1 kit and the same primers as used for the PCR reaction on an ABI Prism 3130xl Genetic Analyzer (Applied Biosystems, Foster City, CA). Sequences (obtained in both directions) were analyzed by comparing them with the germline *GATA1* exon 2 sequence (Accession number NT_011568).

**Supplemental file 3**

***GATA1* targeted deep sequencing (TDS)**

Viable cells from neonates with DS were thawed and DNA was extracted using the Qiagen AllPrep RNA/DNA extraction kit. A two-step PCR of exon 2 and 3 of *GATA1* was performed. The details of the *GATA1* exon 2 and 3 TDS are included in supplemental file 4. All variants detected were validated, to rule out false positive results. The detection limit of this method was defined using a serial dilution of gDNA from the *GATA1* positive cell line CMK of 10%, 2%, 1%, 0.5%, 0.2%, 0.1% and 0.01% in gDNA from CMS (*GATA1* negative). At 0.01%, no GATA1 mutation was detected (Supplemental figure 1). For the exon 3 primer set no positive control was available so we were unable to define the detection limit of this primer pair.

**Supplemental file 4**

***GATA1* next generation sequencing (NGS)**

NGS can detect mutations at much lower frequencies compared to conventional techniques. The technique described here, can detect mutations with a frequency as low as 0.1%. To create a sequencing library for NGS, a two-step PCR has to be performed. In the first step, a PCR product with a tag sequence is generated. As mutations in *GATA1* occur in exon 2 and 3, two primer pairs were designed:

*GATA1* exon 2 Fw TCGTCGGCAGCGTCAGATGTGTATAAGAGACAGGGGAAGGATTTCTGTGTCT; *GATA1* exon 2 Rv GTCTCGTGGGCTCGGAGATGTGTATAAGAGACAGGACCTAGCCAAGGATCTCCATGG

*GATA1* exon 3 Fw TCGTCGGCAGCGTCAGATGTGTATAAGAGACAGGTGCGCTGACCCTAGACTG; *GATA1* exon 3 Rv GTCTCGTGGGCTCGGAGATGTGTATAAGAGACAGCCGGTGGGAGAAAAGAAGGTA.

The tag sequence is used as a priming site for a second round of limited cycle PCR to introduce the sequencing priming sites, indexes and p5 and p7 adapter sequences. After the limited cycle PCR, the amplicons are sequenced using the Illumina MiSeq system. Human genome 38 (CRCh38) is used as the reference sequence. Alignment to CRCh38 genome and variant calling is performed by CLCbio Genomics Workbench. Variant effect prediction analysis is performed. Variants with a frequency less than 1% are validated, to rule out false positive results.

**Supplemental figure 1**

***GATA1* mutation TDS detection threshold**

Serial dilution of gDNA from CMY diluted in gDNA from CMS, to define the detection threshold of *GATA1* mutations using TDS. At 0.01% dilution, no *GATA1* mutation was detected.
